# Risk Factors for Mortality Associated With Influenza Infection in Patients With Hematologic Malignancies: A Cross‐Sectional Descriptive Observational Study

**DOI:** 10.1002/hsr2.72898

**Published:** 2026-07-25

**Authors:** Setayesh Sindarreh, Seyed Amirhossein Dormiani Tabatabaei, Mehran Sharifi, Maryam Nasirian, Seyed Hamed Tooyserkani, Fariba Alikhani, Hamide Rahmani Seraji, Arefeh Zamani, Atousa Hakamifard

**Affiliations:** ^1^ Cancer Prevention Research Center Isfahan University of Medical Sciences Isfahan Iran; ^2^ Department of Biostatistics and Epidemiology, School of Health Isfahan University of Medical Sciences Isfahan Iran; ^3^ School of Medicine Isfahan University of Medical Sciences Isfahan Iran; ^4^ Department of Radiology, School of Medicine Isfahan University of Medical Sciences Isfahan Iran; ^5^ Department of Hematology and Oncology, Taleghani Hospital Shahid Beheshti University of Medical Sciences Tehran Iran; ^6^ Student Research Committee Isfahan University of Medical Sciences Isfahan Iran; ^7^ Infectious Diseases and Tropical Medicine Research Center Isfahan University of Medical Sciences Isfahan Iran

## Abstract

**Background and Aims:**

Cancer patients, especially those with hematologic malignancies (HMs), are considered among the most vulnerable groups at risk for severe outcomes from influenza infection. This study aimed to investigate influenza infection in patients with HMs and identify the risk factors associated with mortality.

**Methods:**

This cross‐sectional descriptive observational study included 90 patients with confirmed influenza infection and HMs. The data collected included demographic information, cancer type and status, influenza type, symptoms, laboratory and radiologic findings, length of hospital stay, ICU admissions, and mortality rate. The primary outcome was analysis endpoints included mortality risk factors based on ICU admission, death and type of malignancies.

**Results:**

The mean age of patients was 34.3 ± 26.7 years (median 34, range 2–91). Most had acute lymphoblastic leukemia (ALL, 45.6%), followed by acute myeloid leukemia (AML, 22.2%), lymphomas (14.4%), multiple myeloma (MM, 6.7%), chronic myelogenous leukemia (CML, 2.2%), and other malignancies (8.9%). Nearly half (49.3%) were in the maintenance phase of treatment, and 80% were infected with influenza A. Pleural effusion (22.2%) and ground‐glass opacities (17.8%) were the most common radiologic findings. ICU admission was significantly linked to older age (*p* = 0.002), comorbidity (*p* = 0.003), lower respiratory tract infection (LRTI) (*p* = 0.02), muscle ache (*p* = 0.03), and malignancies including MM, CML, and AML (*p* = 0.02). Higher mortality was observed in patients with MM or AML (*p* = 0.01), comorbidities (*p* = 0.02), and LRTIs (*p* = 0.04). Age‐adjusted analysis confirmed MM, LRTI, and comorbidity as potential strong predictors of mortality.

**Conclusion:**

ICU admission was significantly associated with older age, the presence of at least one comorbidity, LRTI, and body muscle aches. MM, CML, and AML were notably more associated with ICU admission. Patients diagnosed with MM and AML, those with at least one comorbidity, and those with LRTIs had a higher risk of mortality. The age‐adjusted analysis identified MM, LRTIs, and comorbidities as potential strong predictors of mortality in this cohort, requiring validation in larger studies.

## 1 Introduction

Influenza, commonly known as the flu, is a highly contagious respiratory illness primarily caused by influenza A and B viruses. The global burden of influenza infections has raised significant healthcare concerns, as seasonal epidemics lead to substantial morbidity and mortality. The virus's rapid spread during the influenza season poses significant public health challenges [1, 2, 3]. Influenza is typically characterized by a symptom complex that includes fever, cough, sore throat, myalgia, and fatigue [4]. However, the disease presents an even greater risk for severe complications in vulnerable individuals. Pre‐existing medical conditions or other factors that compromise immune function notably increase one's risk [5]. Age, comorbidities, and immunosuppressive status further exacerbate this vulnerability. As such, a comprehensive analysis of the impact of influenza on a highly susceptible population, such as cancer patients, from both clinical and virological perspectives is crucial [6, 7, 8, 9].

Among vulnerable groups, cancer patients are recognized as one of the highest‐risk populations [9]. These patients often have significantly impaired immune responses due to both their underlying malignancies and the aggressive therapies they typically undergo. Treatments like chemotherapy and radiation therapy, while essential for treating cancer, can further compromise the immune system, making patients more susceptible to infections and other complications [10]. Cancer patients are at increased risk of influenza infection not only because of their weakened immune systems but also due to the effects of their underlying diseases. These patients often experience higher incidence rates, morbidity, and, importantly, mortality associated with influenza [10]. The study by El Ramahi and Freifeld highlights the increased susceptibility of cancer patients to influenza due to their compromised immune systems, resulting from both the cancer itself and the immunosuppressive treatments they receive [11].

While malignancy is widely recognized as a factor that negatively impacts the severity of influenza, there is a notable gap in the literature regarding the clinical outcomes of patients with hematologic malignancies (HMs) affected by this virus. This lack of data underscores the need for further research into how HMs influence the progression and management of influenza infections. Understanding these dynamics is crucial for improving patient care and outcomes in this vulnerable population. Therefore, this study aims to investigate influenza infection in patients with HMs and identify the risk factors associated with its mortality.

## 2 Methods

This was a cross‐sectional descriptive observational study approved by the ethical code IR.ARI.MUI.REC.1403.069 at Isfahan University of Medical Sciences. The study population consisted of patients with HMs referred to Omid Hospital, a referral cancer center, during the influenza seasons, which spanned September 2022 to March 2024. Written informed consent was obtained from all the patients.

Patients were included in the study if they met the following criteria: any age with a confirmed diagnosis of HM and a positive influenza real‐time polymerase chain reaction (PCR) test result. Exclusion criteria included incomplete medical records, a history of immunosuppressive diseases unrelated to cancer, hematopoietic stem‐cell transplantation (HSCT) recipients, and the diagnosis of another concurrent infection.

To collect the required data, the researchers initially contacted the infection control unit to obtain a list of patients diagnosed with influenza over the past 2 years. Next, only those with a definitive diagnosis of influenza, confirmed by a positive PCR test for either type A or B influenza virus, were included in the study. It was crucial to distinguish influenza cases from COVID‐19 cases, ensuring that only influenza‐positive patients were considered for inclusion.

The names and file numbers of eligible participants were retrieved from the lists maintained by the infection control unit. Additional data were then gathered using a checklist developed by the researchers, which included demographic information such as gender, age, height, and weight, as well as clinical details regarding the type and status of cancer, type of influenza, respiratory tract infection (upper or lower), disease symptoms, laboratory findings, comorbidities, length of hospitalization, and outcomes such as ICU admissions and mortality. In this study, lower respiratory tract infection (LRTI) was defined based on the presence of parenchymal involvement revealed in the lung CT scan. Oseltamivir was initiated in all hospitalized patients, and according to medical records, it was started within 48 h of symptom onset in the majority of cases. However, precise adherence to this window could not be verified retrospectively for every patient.

It should also be noted that, for the purpose of statistical analysis and improved clinical interpretation, continuous variables—including weight‐related measures, laboratory values, and vital signs—were categorized into dichotomous variables based on established clinical cutoffs. These cutoffs were selected from widely accepted guidelines and standard clinical practice.

Data were collected from patient files and the hospital's data recording system (Hospital Information System, HIS). In cases where information was missing, the data were supplemented by inpatient departments. Patients with more than 30% incomplete data in their medical files were excluded from the study.

### 2.1 Outcome Assessment

Outcome assessment focused on two main endpoints: in‐hospital mortality and ICU admission. In‐hospital mortality was defined as death occurring during the index hospitalization for influenza infection. ICU admission was defined as transfer to the intensive care unit at any point during hospitalization.

### 2.2 Statistical Analysis

Following data collection, the data were entered into SPSS version 23.0 and checked for missing values and outliers. Descriptive statistics were used to summarize baseline characteristics, including mean ± standard deviation (SD) or median with interquartile range (IQR) for continuous variables, and frequency (percentage) for categorical variables. Comparisons between groups were made using the *χ* ^2^ test or Fisher's exact test for categorical variables, and Student's *t*‐test or Mann–Whitney U test for parametric and nonparametric continuous variables, respectively [12]. It should be noted that percentages were calculated based on patients with complete data for each variable (available‐case analysis), meaning denominators represent only cases without missing values for the specific measure being analyzed. To identify independent predictors of mortality and ICU admission, logistic regression models were fitted separately for adults and children. Additionally, with age adjustment, logistic regression was used to evaluate the impact of risk factors on mortality and ICU admission. Odds ratios (ORs) and 95% confidence intervals (CIs) were calculated to assess the strength of associations [13]. All statistical tests were two‐sided, and a *p* value < 0.05 was considered statistically significant [14].

## 3 Results

### 3.1 Baseline Characteristics

A total of 90 patients with laboratory‐confirmed influenza were identified at a cancer center during the peak flu season. Of these, 59 patients (65.6%) were male. The mean age was 34.29 ± 26.73 years (median 34, range 2–91 years).

Of the 90 patients, 41 (45.6%) had acute lymphoblastic leukemia (ALL), and 20 (22.2%) had acute myeloid leukemia (AML), 13 (14.4%) had lymphomas, 6 (6.7%) had Multiple Myeloma (MM), 2 (2.2%) had Chronic Myelogenous Leukemia (CML), and 8 patients (8.9%) had other types of malignancies. In patients under 18 years old, the majority (80.6%) had ALL. However, only 22.2% of patients over 18 were classified as ALL, and this disparity was statistically significant (*p *< 0.001). Table 1 shows the demographic and clinical characteristics of the participants, including their cancer types and underlying diseases.

**Table 1:** Demographic and clinical characteristics of cancer and underlying diseases of the participants.

Variables	All patients *N* = 90	≥ 18 years *N* = 54	< 18 years *N* = 36	*p* value
Age^a^ [median (IQR)]	30 (6, 55.5)	53 (36,68)	6 (4,8)	*p* < 0.001
Weight^a^ [median (IQR)]	60 (22.5, 70.5)	69 (61,75)	23 (18.25, 37)	*p* < 0.001
Height^a^ [median (IQR)]	165 (116.5, 170)	168 (162, 175)	111 (97.5, 143.5)	*p* < 0.001
BMI^a^ [mean ± SD]	22.32 ± 5.49	23.89 ± 5.4	20.06 ± 4.89	0.003
Obesity		*N* (%^b^)		
No (BMI < 30)	72 (90)	42 (85.7)	30 (96.8)	*p* = 0.11
Yes (BMI ≥ 30)	8 (10)	7 (14.3)	1 (3.2)	
Gender		*N* (%^b^)		
Male	59 (65.6)	37 (68.5)	22 (61.1)	*p* = 0.50
Female	31 (34.4)	17 (31.5)	14 (38.9)	
Types of malignancy		*N* (%^b^)		
ALL	41 (45.6)	12 (22.2)	29 (80.6)	
AML	20 (22.2)	18 (33.3.)	2 (5.6)	
CML	2 (2.2)	2 (2.2)	0 (0)	*p* < 0.001
Lymphomas	13 (14.4)	10 (18.5)	3 (8.3)	
MM	6 (6.7)	5 (9.3)	1 (2.8)	
Other	8 (8.9)	1 (7.8)	1 (2.8)	
Cancer status		*N* (%^b^)		
Consolidation	24 (32)	12 (37.5)	12 (27.9)	
Induction	7 (9.3)	5 (15.6)	2 (4.7)	*p* = 0.21
Maintenance	37 (49.3)	12 (37.5)	25 (58.1)	
Refractory	7 (9.3)	3 (9.4)	4 (9.3)	
Chemotherapy during flu		*N* (%^b^)		
Yes	46 (54.1)	24 (49)	22 (61.1)	*p* = 0.28
No	39 (45.9)	25 (51)	14 (38.9)	
Comorbidities^c^		*N* (%^b^)		
DM	4 (4.4)	4 (7.4)	0 (0)	*p* = 0.15
HTN	3 (3.3)	3 (5.6)	0 (0)	*p* = 0.27
IHD	1 (1.1)	1 (1.9)	0 (0)	*p* = 0.41
CKD	0 (0)	0 (0)	0 (0)	—
ESDR	0 (0)	0 (0)	0 (0)	—
Other	7 (7.8)	7 (12.96)	0 (0)	*p* = 0.15

^a^ Variables were tested for normality; non‐normally distributed variables are expressed as median with interquartile range (IQR), while normally distributed variables are expressed as mean ± SD.

^b^ Percentages are calculated using valid denominators after excluding missing values. For example, out of 90 patients, cancer status was documented for 75 patients, Chemotherapy during flu was documented for 85 patients, and BMI was documented for 80 patients.

^c^ The percentage of positive cases of each disease is listed.

Out of 75 patients with known cancer status, 37 (49.3%) were in the maintenance phase of treatment. Additionally, more than half of the patients (54.1%) underwent chemotherapy. Thirteen patients (14.4%) had at least one underlying disease. The data in Table 1 show that no patients under 18 had any underlying diseases.

Table 2 presents the clinical characteristics of influenza in these patients. According to the data, most patients (80%) were affected by type A influenza. Among 90 patients, 49 (54.4%) presented with lower respiratory tract infections (LRI), while 41 (45.6%) had upper respiratory tract infections (URI). The most common symptoms were fever (74.4%), followed by cough (34.4%) and body muscle aches (33.3%). The results indicate that cough and body pain were significantly more common in adults (over 18 years old).

**Table 2:** Clinical characteristics of influenza in participants.

Variables		All patients *N* = 90	≥ 18 years *N* = 54	< 18 years *N* = 36	*p* value
Type of influenza [N* = 90]			*N* (%)		
A		72 (80)	42 (77.8)	30 (83.3)	*p* = 0.69
B		18 (20)	12 (22.2)	6 (16.7)	
Site of infection [N* = 90]			*N* (%)		
LRI		49 (54.45)	39 (72.22)	10 (27.8)	*p* < 0.001
URI		41(45.55)	15 (27.77)	26 (66.7)	
Symptoms^a^		*N* (%)			
Fever		67 (74.4)	42 (77.8)	25 (69.4)	*p* = 0.46
Cough		31 (34.4)	24 (44.4)	7 (19.4)	*p* = 0.02
Body muscle ache		30 (33.3)	24 (44.4)	6 (16.7)	*p* = 0.007
Weakness/fatigue		49 (54.4)	33 (61.1)	16 (32.7)	*p* = 0.13
Headache		6 (6.7)	5 (9.3)	1 (2.8)	*p* = 0.39
Chills		15 (16.7)	12 (22.2)	3 (8.3)	*p* = 0.15
Dyspnea		11 (12.2)	9 (16.7)	2 (5.6)	*p* = 0.19
Diarrhea		2 (2.2)	1 (1.9)	1 (2.8)	*p* = 0.77
Sore throat		12 (13.3)	9 (16.7)	3 (8.3)	*p* = 0.35
Others		22 (24.4)	12 (22.2)	10 (27.8)	*p* = 0.62
Vital signs at admission^a^		[Median (IQR)] or [mean ± SD]			
Temperatures		38.28 ± 1.16	38.19 ± 1.07	38.41 ± 1.29	*p* = 0.39
RR		20.73 ± 3.05	20.58 ± 3.2	20.94 ± 2.86	*p* = 0.60
PR		115.27 ± 26.47	104.84 ± 25.78	130.17 ± 19.61	*p* < 0.001
Systolic bp		109.5 (99, 126.25)	120 (106, 134.25)	99.5 (90, 105)	*p* < 0.001
Diastolic bp		70 (59.25, 80)	77 (67.75, 80)	60 (50,70)	*p* < 0.001
O2sat		96 (92,97)	94 (92,96)	96.5 (96,98)	*p* < 0.001
Vital signs at admission categories			*N* (%)		
Temperatures	Normal	27 (30)	18 (33.3)	9 (25)	*p *= 0.49
[N* = 90]	Fever	63 (70)	36 (66.7)	27 (75)	
RR	Normal	42 (46.7)	14 (25.9)	28 (77.8)	*p *< 0.001
[N* = 90]	Tachypnea	48 (53.3)	40 (74.1)	8 (22.2)	
PR	Normal	36 (40)	22 (40.7)	14 (38.9)	*p *= 0.86
[N* = 90]	Tachycardia	54 (60)	32 (59.3)	22 (61.1)	
Blood pressure	Normal	69 (76.7)	37 (68.5)	32 (88.9)	*p *= 0.04
[N* = 90]	Hypertension	21 (23.3)	17 (31.5)	4 (11.1)	
O2sat	Normal	62 (72.1)	32 (61.5)	30 (88.2)	*p *= 0.007
[N* = 90]	Hypoxemia	24 (27.9)	20 (38.5)	4 (11.8)	
Laboratory findings^a^		[Median (IQR)] or [mean ± SD]			
Neutrophil		32600 (3175, 71975)	11000 (2950, 71400)	37300 (3000, 73000)	*p* = 0.72
Lymphocyte		8450 (1175, 21025)	6000 (1200, 14400)	9200 (800, 32500)	*p* = 0.15
Cr		1.13 ± 0.64	1.23 ± 0.7	0.96 ± 0.48	*p* = 0.07
BUN		17.23 ± 12.08	20.65 ± 12.58	10.86 ± 7.93	*p* < 0.001
CRP		86 (37, 114)	97 (58, 120)	53 (5, 102)	*p* = 0.01
AST		33 (22.5, 52.5)	33 (17,63)	33 (24,46)	*p* = 0.37
ALT		18.5 (13.75, 48.75)	21.5 (13, 51.75)	17.5 (14, 40.25)	*p* = 0.97
LDH		600 (471, 756)	602 (434, 852)	588.5 (504.5, 693)	*p* = 0.57
Laboratory findings categories			*N* (%)		
Neutrophil	Normal	31 (47)	18 (51.4)	13 (41.9)	*p *= 0.46
[N* = 66]	Abnormal	35 (53)	17 (48.6)	18 (58.1)	
Lymphocyte	Normal	19 (27.5)	13 (34.2)	6 (18.4)	*p *= 0.18
[N* = 69]	Abnormal	50 (72.5)	25 (65.8)	25 (80.6)	
Cr	Normal	62 (74.7)	38 (74.5)	24 (75)	*p *= 0.96
[N* = 83]	Abnormal	21 (25.3)	13 (25.5)	8 (25)	
BUN	Normal	67 (76.1)	33 (63.5)	34 (94.4)	*p *= 0.001
[N* = 88]	Abnormal	21 (23.9)	19 (36.5)	2 (5.6)	
CRP	Normal	16 (25.4)	7 (17.5)	9 (39.1)	*p *= 0.07
[N* = 63]	Abnormal	47 (74.6)	33 (82.5)	14 (60.9)	
AST	Normal	51 (66.2)	27 (60)	24 (75)	*p *= 0.22
[N* = 77]	Abnormal	26 (33.8)	18 (40)	8 (25)	
ALT	Normal	54 (73)	30 (68.2)	24 (80)	*p *= 0.29
[N* = 74]	Abnormal	20 (27)	14 (31.8)	6 (20)	
LDH	Normal	11 (21.2)	10 (25)	1 (8.3)	*p *= 0.42
[N* = 52]	Abnormal	41 (78.8)	30 (75)	11 (91.7)	
CT scan findings N* = 65			*N* (%)		
Nodules		12 (13.3)	9 (16.7)	3 (8.3)	*p* = 0.35
Pleural effusion		20 (22.2)	18 (33.3)	2 (5.6)	*p* = 0.002
Consolidation		9 (10)	7 (13)	2 (5.6)	*p* = 0.30
Cardiomegaly		1 (1.1)	1 (1.9)	0(0)	*p* = 0.41
GGO		16 (17.8)	14 (25.9)	2 (5.6)	*p* = 0.02
Other		7 (7.8)	5 (9.3)	2 (5.6)	*p* = 0.7
Duration of hospitalization [median (IQR)]		8 (5,8)	8 (5,8)	7 (4,7)	*p* = 0.53

Note: N*: The number of observations for each variable.

^a^ Variables were tested for normality; non‐normally distributed variables are expressed as median with interquartile range (IQR), while normally distributed variables are expressed as mean ± SD.

In terms of vital sign categories, tachypnea was more common in adults (74.1% vs. 22.2%; *p* < 0.001), hypertension was also more prevalent among adults (31.5% vs. 11.1%; *p* = 0.04), and hypoxemia was more frequent in adults compared to children (38.5% vs. 11.8%; *p* = 0.007).

Laboratory findings revealed that adults had significant BUN levels compared to children (20.65 ± 12.58 vs. 10.86 ± 7.93; *p* < 0.001), while children were more likely to have normal BUN levels (94.4% vs. 63.5%; *p* = 0.001). C‐reactive protein (CRP) levels were elevated in both groups, though not statistically different (*p* = 0.07). No significant differences were observed in neutrophil or lymphocyte counts between the two age groups.

Additionally, CT scan results revealed that pleural effusion (22.2%) and ground‐glass opacities (GGO) (17.8%) were the most common findings among patients. These findings were notably more prevalent in adults compared to those under 18 years old (Table 2).

### 3.2 Outcome Analysis

The outcomes of influenza were investigated, revealing that a total of 9 patients (10%) died, and 13 patients (14.4%) were admitted to the intensive care unit (ICU).

The median (Q1, Q3) duration of hospitalization was 8 [5, 8] days, with a range of 1–62 days.

Furthermore, the clinical outcomes were compared according to the study variables, with the findings presented in Table 3. The results show that patients aged over 41 years had significantly higher mortality than younger patients (8 out of 36 vs. 1 out of 54; *p* = 0.04), using Fisher's exact test.

**Table 3:** Outcomes of the influenza (mortality and ICU admission) according to the variables.

Patients based on research variables	Died *N* (%)		*p* value	ICU admission *N* (%)		*p* value
	Yes	No		Yes	No	
All patients	9 (10)	81 (90)		13 (14.4)	77 (85.6)	
Gender			*p* = 0.94			*p* = 0.76
Male	6 (10.2)	53 (89.8)		9 (15.3)	50 (84.7)	
Female	3 (9.7)	28 (90.3)		4 (12.9)	27 (87.1)	
Age			*p* = 0.08			*p* = 0.06
< 18 years	1 (2.8)	35 (97.2)		2 (5.6)	34 (94.4)	
≥ 18 years	8 (14.8)	46 (85.2)		11 (20.4)	43 (79.6)	
Age category			*p* = 0.04			*p* = 0.002
< 18 years	1 (2.8)	35 (97.2)		2 (5.6)	34 (94.4)	
18–30 years	0 (0)	5 (100)		0 (0)	5 (100)	
31–40 years	0 (0)	13 (100)		0 (0)	13 (100)	
41–60 years	4 (24)	12 (75)		3 (18.8)	13 (81.2)	
> 60 years	49 (20)	16 (80)		8 (40)	12 (60)	
Types of malignancy			*p* = 0.01			*p* = 0.02
ALL	2 (4.9)	39 (95.1)		2 (4.9)	39 (95.1)	
AML	3 (15)	17 (85)		5 (25)	15 (75)	
CML	0 (0)	2 (100)		1 (50)	1 (50)	
Lymphomas	0 (0)	13 (100)		1 (7.7)	12 (92.3)	
MM	3 (50)	3 (50)		3 (50)	3 (50)	
Other	1 (12.5)	7 (87.5)		1 (12.5)	7 (87.5)	
Cancer status			*p* = 0.20			*p* = 0.18
Consolidation	0 (0)	24 (34.8)		22 (33.8)	2 (20)	
Induction	0 (0)	7 (10.1)		7 (10.8)	0 (0)	
Maintenance	5 (83.3)	32 (46.4)		29 (44.6)	8 (80)	
Refractory	1 (16.7)	6 (8.7)		7 (10.8)	0 (0)	
Chemotherapy			*p* = 0.54			*p* = 0.55
Yes	4 (8.7)	42 (91.3)		5 (10.9)	41 (89.1)	
No	5 (12.8)	34 (87.2)		6 (15.4)	33 (84.6)	
At least one comorbidity			*p* = 0.02			*p* = 0.003
Yes	4 (30.8)	9 (69.2)		6 (46.2)	7 (53.8)	
No	5 (6.5)	72 (93.5)		7 (9.1)	90 (90.9)	
Type of influenza			*p* = 0.05			*p* = 0.12
A	5 (6.9)	67 (93.1)		8 (11.1)	64 (88.9)	
B	4 (22.2)	14 (77.8)		5 (27.8)	13 (72.2)	
Site of infection			*p* = 0.04			*p* = 0.02
LRI	8 (16.3)	41 (83.7)		11 (22.4)	38 (77.6)	
URI	1 (2.4)	40 (97.6)		2 (4.9)	39 (95.1)	
Fever			*p* = 0.57			*p* = 0.64
Yes	6 (9)	61 (91)		9 (13.4)	58 (86.6)	
No	3 (13)	20 (87)		4 (17.4)	19 (82.6)	
Cough			*p* = 0.71			*p* = 0.76
Yes	2 (6.5)	29 (93.5)		4 (12.9)	27 (87.1)	
No	7 (11.9)	52 (88.1)		9 (15.3)	50 (84.7)	
Body muscle ache			*p* = 0.15			*p* = 0.03
Yes	5 (16.7)	25 (83.3)		8 (26.7)	22 (73.3)	
No	4 (6.7)	56 (93.3)		5 (8.3)	55 (91.7)	
Weakness/fatigue			*p* = 0.94			*p* = 0.56
Yes	5 (10.2)	44 (89.8)		6 (12.2)	43 (87.8)	
No	4 (9.8)	37 (90.2)		7 (17.1)	34 (82.9)	

Patients diagnosed with MM and AML experienced notably higher mortality rates compared to other types of malignancies (*p* = 0.01). Moreover, patients with at least one comorbidity exhibited significantly higher mortality rates (*p* = 0.02). However, no significant relationship was found between chemotherapy (*p* = 0.54), cancer status (*p* = 0.20), and mortality rates.

Although patients with influenza type B had a higher mortality rate, there was no statistically significant difference between the two groups (*p *= 0.05). The results indicate that lower respiratory infection (LRI) is substantially associated with higher mortality (*p *= 0.04). However, none of the influenza symptoms were linked to increased mortality (Table 3).

On the other hand, ICU admission was significantly associated with older age (over 41 years old) (11 out of 54 adults vs. 2 out of 36 children; *p* = 0.002), and the presence of at least one comorbidity (6 out of 13 ICU patients vs. 7 out of 77 non‐ICU patients; *p* = 0.003).

Also, 22.4% of patients with LRI required ICU care, compared to only 4.87% of those with URI (*p* = 0.02). Body muscle ache was significantly more common among patients admitted to the ICU (26.7% vs. 8.3%, *p* = 0.03). Among cancer types, MM (50%), CML (50%), and AML (25%) were significantly more likely to be associated with ICU admission (*p* = 0.02).

Furthermore, in multivariable logistic regression analysis adjusted for age, several factors were significantly associated with adverse outcomes among patients with influenza. Patients with multiple myeloma had significantly increased odds of death compared to those with ALL (OR = 35.32, 95% CI: 2.09–95.72). The presence of at least one comorbidity was also significantly associated with higher odds of death (OR = 4.125, 95% CI: 1.111–11.36). URI infection showed a protective effect against mortality (OR = 0.291, 95% CI: 0.029–0.936).

For ICU admission, although most hematologic malignancies showed elevated odds, only URI infection reached statistical significance (OR = 7.203, 95% CI: 1.77–66.71). Patients with MM had the highest odds of ICU admission (OR = 52.39, 95% CI: 2.92–939.65), though the wide confidence interval suggests uncertainty due to small sample size in this subgroup (Table 4).

**Table 4:** Factors affecting death and ICU admission.

Variable	Death			ICU admission		
	OR	95% CI		OR	95% CI	
		Lower	Upper		Lower	Upper
Types of malignancy^b^						
ALL	Ref	Ref	Ref	Ref	Ref	Ref
AML	2.76	0.17	44.23	10.69	0.78	14.68
CML	—	—	—	34.52	1.82	140.08
Lymphomas	—	—	—	3.18	0.15	65.54
MM	35.32^a^	2.09	95.72	52.39	2.92	939.65
Cancer status^b^						
Consolidation	Ref	Ref	Ref	Ref	Ref	Ref
Maintenance	1.26	0.08	8.93	2.65	0.5	14.1
Chemotherapy (yes)	2.027	0.339	12.11	1.755	0.381	8.09
At least one comorbidity (yes)	4.125^a^	1.111	11.36	2.657	0.510	13.83
Type of influenza						
A	Ref	Ref	Ref	Ref	Ref	Ref
B	3.892	0.427	35.54	3.221	0.667	15.56
Site of infection						
LRI	Ref	Ref	Ref	Ref	Ref	Ref
URI	0.291^a^	0.029	0.936	0.118^a^	0.014	0.994
Fever (yes)	1.433	0.152	13.49	0.860	0.161	4.59
Cough (yes)	0.614	0.1	3.77	1.13	0.276	4.63
Body muscle ache (yes)	1.338	0.218	8.23	1.817	0.453	7.29
Weakness/fatigue (yes)	1.44	0.263	7.91	1.44	0.368	5.44

*Note:* Wide confidence intervals for some subgroups (e.g., MM, CML) reflect small sample sizes; these estimates should be interpreted with caution.

^a^ Significant.

^b^ Other categories not shown in the table were excluded from the final model due to the absence of outcome events or non‐convergence (e.g., empty cells or insufficient data).

## 4 Discussion

In the current study, we described the clinical presentations and outcomes of influenza infection in patients with HMs who developed influenza during influenza seasons at a referral cancer center. Although respiratory viruses typically cause self‐limiting infections in the general population, patients with underlying HMs are particularly vulnerable to influenza complications and in‐hospital mortality [10]. Chemotherapy notably impacts the body's immune defense mechanisms, thereby weakening them. This may explain the recurring outbreaks of influenza observed in hematology departments over the years [15, 16].

Most of the patients were infected with type A influenza, which affected all age groups. Our findings align with other studies that report fever and cough as the most frequent symptoms in cancer patients [17]. Among the patients, those with AML and MM had the highest influenza‐related mortality rates. Additionally, the rate of ICU admission was higher in patients with AML, MM, and CML. Although patients with influenza type B had a higher mortality rate, our results indicate that LRTIs are notably associated with higher mortality, regardless of the influenza type.

Older age and comorbidities were significant risk factors for severe influenza outcomes. These findings are consistent with recent studies, emphasizing the importance of implementing effective strategies to prevent, diagnose, and treat influenza in this vulnerable population [18].

Our study showed that the most common CT scan finding was pleural effusion, followed by GGOs. Several studies have shown that pleural effusion in influenza patients is mainly associated with severe infection requiring ICU care [19, 20, 21].

Considering the differences in chemotherapy between adults and children, we evaluated an age‐based comparison between the two groups. No significant difference was shown between these groups. This revealed that MM, LRTIs, and comorbidities were strong predictors of patient mortality.

Our study has several limitations. The retrospective design and relatively small sample size may limit the generalizability of the findings. The strong age‐cancer subtype association (pediatric ALL vs. adult AML/MM) limits disentangling their independent effects. Additionally, small subgroups (e.g., MM, CML) resulted in wide confidence intervals; thus, findings from these subgroups should be considered exploratory rather than confirmatory. Future age‐stratified, multi‐center studies are needed. Additionally, the lack of a control group of healthy individuals prevents direct comparisons of influenza severity between cancer patients and the general population.

Additionally, we could not collect data on influenza vaccination status or the precise attribution of death to influenza versus other competing causes (e.g., induction therapy complications or malignancy progression), which may have influenced the outcomes.

Future research should focus on evaluating the effectiveness of novel preventive strategies, especially in high‐risk patients. Prospective studies with larger sample sizes are needed to further explore influenza's risk factors and outcomes in this vulnerable population. These studies should also consider collecting vaccination status data to better assess its impact on clinical outcomes in patients with hematologic malignancies.

## 5 Conclusion

This study examines the epidemiology, risk factors, outcomes, and predictors of mortality related to influenza infection among patients with HMs. Older age, the presence of at least one comorbidity, LRTIs, body muscle aches, as well as MM, AML, and CML, were substantially associated with ICU admission. Patients diagnosed with MM and AML, along with those having at least one comorbidity and LRTIs, were more likely to experience higher mortality. The age‐adjusted analysis further identified MM, LRTIs, and having at least one comorbidity as strong predictors of mortality.

## Author Contributions

**Setayesh Sindarreh:** formal analysis, data curation, writing – original draft. **Seyed Amirhossein Dormiani Tabatabaei:** data curation, writing – original draft, investigation. **Mehran Sharifi:** writing – review and editing, validation, conceptualization. **Maryam Nasirian:** methodology, formal analysis. **Seyed Hamed Tooyserkani:** data curation, investigation. **Fariba Alikhani:** data curation. **Hamide Rahmani Seraji:** investigation, conceptualization. **Arefeh Zamani:** data curation. **Atousa Hakamifard:** conceptualization, validation, visualization, writing – review and editing, supervision.

## Consent

Written informed consent was obtained from all the participants.

## Conflicts of Interest

The authors declare no conflicts of interest.

## Transparency Statement

The corresponding author, Atousa Hakamifard, affirms that this manuscript is an honest, accurate, and transparent account of the study being reported; that no important aspects of the study have been omitted; and that any discrepancies from the study as planned (and, if relevant, registered) have been explained.

## Data Availability

The data that support the findings of this study are available from the corresponding author upon reasonable request.

## References

[1] X. Wang , Y. Li , K. L. O'Brien , et al., “Global Burden of Respiratory Infections Associated With Seasonal Influenza in Children Under 5 Years in 2018: A Systematic Review and Modelling Study,” Lancet Global Health 8, no. 4 (2020): e497–e510. 32087815 10.1016/S2214-109X(19)30545-5 PMC7083228

[2] Y. Gao , M. Liu , K. Numata , et al., “Global and National Influenza‐Associated Hospitalization and Mortality Rates: A Systematic Review and Meta‐Analysis,” medRxiv 8, no. 8 (2025): 25333141, 10.1101/2025.08.08.25333141.

[3] J. Paget , L. Staadegaard , X. Wang , et al., “Global and National Influenza‐Associated Hospitalisation Rates: Estimates for 40 Countries and Administrative Regions,” Journal of Global Health 13 (2023): 04003. 36701368 10.7189/jogh.13.04003 PMC9879557

[4] M. H. Ebell , I. Rahmatullah , C. Hulme , et al., “Accuracy of Individual Signs and Symptoms and Case Definitions for the Diagnosis of Influenza in Different Age Groups: A Systematic Review With Meta‐Analysis,” BMJ Open 15, no. 3 (2025): e067574. 10.1136/bmjopen-2022-067574 PMC11877276 40032401

[5] W. P. Hanage and W. Schaffner , “Burden of Acute Respiratory Infections Caused by Influenza Virus, Respiratory Syncytial Virus, and SARS‐CoV‐2 With Consideration of Older Adults: A Narrative Review,” supplement, Infectious Diseases and Therapy 14, no. Suppl 1 (2025): 5–37. 39739200 10.1007/s40121-024-01080-4 PMC11724833

[6] T. Shafat , D. De‐la‐Rosa‐Martinez , F. Khawaja , et al., “Outcomes and Risk Factors for Influenza and Respiratory Syncytial Virus Lower Respiratory Tract Infections and Mortality in Patients With Lymphoma or Multiple Myeloma: A 7‐Year Retrospective Cohort Study,” Open Forum Infectious Diseases 12, no. 4 (2025). 10.1093/ofid/ofaf127 PMC11961405 40177586

[7] A. Branche , M. Ramesh , and B. Francis , “A Narrative Review of Key Risk Factors for Severe Illness Following SARS‐CoV‐2, Influenza Virus, and Respiratory Syncytial Virus Infection,” Infectious Diseases and Therapy 14, no. S1 (2025): 39–61. 39739198 10.1007/s40121-024-01081-3 PMC11724830

[8] P. Daniels , L. Danziger‐Isakov , and W. Otto , “Seasonal Influenza in Children With Cancer,” Cureus 16, no. 10 (2024): e72785. 39618559 10.7759/cureus.72785 PMC11608092

[9] O. Abdel‐Rahman , “Influenza and Pneumonia‐Attributed Deaths Among Cancer Patients in the United States; a Population‐Based Study,” Expert Review of Respiratory Medicine 15, no. 3 (2021): 393–401. 33107375 10.1080/17476348.2021.1842203

[10] J. Salmanton‐García , F. Marchesi , M. Navrátil , et al., “Respiratory Viruses in Patients With Hematological Malignancy in Boreal Autumn/Winter 2023–2024: EPICOVIDEHA‐EPIFLUEHA Report,” American Journal of Hematology 100, no. 3 (2025): 358–374. 39715069 10.1002/ajh.27565 PMC11803548

[11] R. El Ramahi and A. Freifeld , “Epidemiology, Diagnosis, Treatment, and Prevention of Influenza Infection in Oncology Patients,” Journal of Oncology Practice 15, no. 4 (2019): 177–184. 30970229 10.1200/JOP.18.00567

[12] D. G. Altman , Practical Statistics for Medical Research (Chapman and Hall/CRC, 1990).

[13] E. Vittinghoff , D. V. Glidden , S. C. Shiboski , and C. E. McCulloch , Regression Methods in Biostatistics: Linear, Logistic, Survival, and Repeated Measures Models (Springer Science & Business Media, 2012).

[14] M. Assel , D. Sjoberg , A. Elders , et al., “Guidelines for Reporting of Statistics for Clinical Research in Urology,” Journal of Urology 201, no. 3 (2019): 595–604. 30633111 10.1097/JU.0000000000000001 PMC6600813

[15] J. Li , D. Zhang , Z. Sun , C. Bai , and L. Zhao , “Influenza in Hospitalised Patients With Malignancy: A Propensity Score Matching Analysis,” ESMO Open 5, no. 5 (2020): e000968. 33093022 10.1136/esmoopen-2020-000968 PMC7583803

[16] P. Ljungman , R. de la Camara , L. Perez‐Bercoff , et al., “Outcome of Pandemic H1N1 Infections in Hematopoietic Stem Cell Transplant Recipients,” Haematologica 96, no. 8 (2011): 1231–1235. 21546495 10.3324/haematol.2011.041913 PMC3148919

[17] D. Vilar‐Compte , D. P. Shah , J. Vanichanan , et al., “Influenza in Patients With Hematological Malignancies: Experience at Two Comprehensive Cancer Centers,” Journal of Medical Virology 90, no. 1 (2018): 50–60. 28851056 10.1002/jmv.24930 PMC5761331

[18] F. Mauvais‐Jarvis , “Aging, Male Sex, Obesity, and Metabolic Inflammation Create the Perfect Storm for COVID‐19,” Diabetes 69, no. 9 (2020): 1857–1863. 32669390 10.2337/dbi19-0023 PMC7458034

[19] H.‐Y. Ma , J.‐L. Wu , C.‐Y. Lu , et al., “Risk Factors Associated With Severe Influenza Virus Infections in Hospitalized Children During the 2013 to 2014 Season,” Journal of Microbiology, Immunology and Infection 49, no. 3 (2016): 387–393. 10.1016/j.jmii.2015.05.015 26216185

[20] M. J. Pajor , S. Munigala , D. Reynolds , et al., “Predicting Severe Disease in Patients Diagnosed With Seasonal Influenza in the Emergency Department,” JACEP Open 4, no. 5 (2023): e13045. 37745865 10.1002/emp2.13045 PMC10511830

[21] B. Gu , L. Yao , X. Zhu , P. Tang , and C. Chen , “Comparison of Hospitalized Patients With Severe Pneumonia Caused by COVID‐19 and Influenza A (H7N9 and H1N1): A Retrospective Study From a Designated Hospital,” Open Medicine 17, no. 1 (2022): 1965–1972. 36561841 10.1515/med-2022-0610 PMC9743191

